# A systematic review on the effectiveness of organic unprocessed products in controlling gingivitis in patients undergoing orthodontic treatment with fixed appliances

**DOI:** 10.1002/cre2.417

**Published:** 2021-05-04

**Authors:** Chrysanthi Papadopoulou, Ioanna Karamani, Sofia Gkourtsogianni, Kiriaki Seremidi, Dimitrios Kloukos

**Affiliations:** ^1^ Department of Orthodontics and Dentofacial Orthopedics 251 Hellenic Air Force & VA General Hospital Athens Greece; ^2^ Department of Paediatric Dentistry, Dental School University of Athens Athens Greece; ^3^ Department of Orthodontics and Dentofacial Orthopedics, School of Dental Medicine University of Bern Bern Switzerland

**Keywords:** gingivitis, orthodontic treatment

## Abstract

**Objectives:**

The aim of this systematic review is to summarize the available data on the effects of organic unprocessed products in treating gingivitis during treatment with fixed orthodontic appliances.

**Materials and Methods:**

Multiple electronic databases were searched up to October 1, 2020. Randomized controlled trials (RCTs), controlled clinical trials, cohort studies of prospective and retrospective design, and cross‐sectional studies reporting on natural products for controlling gingivitis in orthodontic patients were eligible for inclusion. The quality of the included RCTs was assessed per the revised Cochrane risk of bias tool for randomized trials (RoB 2.0).

**Results:**

Three RCTs were finally eligible for inclusion, yielding a total of 135 patients with an age range of 12–40 years. Organic products used were *Aloe vera* mouth rinse, ingestion of honey and chamomile mouthwash. Treatment follow‐up period varied from 30 min to 15 days. The results indicated that the use of the aforementioned organic products significantly reduced plaque and gingival bleeding levels as early as treatment started. The reduction in biofilm accumulation and gingival bleeding was significant throughout the studies' follow‐up.

**Conclusions:**

Owing to their antimicrobial and anti‐inflammatory properties, nonpharmacological formulations successfully controlled gingival inflammation and plaque indices in orthodontic patients.

## 1 INTRODUCTION

Gingivitis affects more than 50% of the general population (Yeturu et al., 2016). It is common in all age groups, with increasing prevalence during puberty and peaking between ages 9 and 14 (Martin et al., 2016). Its primary causative factor is poor oral hygiene causing various aerobic and anaerobic bacteria accumulation that form dental biofilms on the teeth and protect the bacteria housed within (Yeturu et al., 2016). It is highly related to increased mechanical plaque retention associated with fixed orthodontic appliances which in turn, increases the rates of periodontal inflammation among orthodontic patients (Martin et al., 2016). While plaque‐induced gingivitis is one of the most usual inflammatory diseases, several non‐plaque‐induced gingival diseases are less common but often of major significance. The non‐plaque‐induced gingival lesions are often manifestations of systemic conditions, but they may also represent pathologic changes limited to gingival tissues.

Orthodontic appliances significantly alter the oral environment and make mechanical removal of plaque difficult for orthodontic patients, who frequently fail to floss and brush properly in the presence of orthodontic archwires (Goes et al., 2016). Plaque build‐up and concomitant gingivitis are increased over the duration of orthodontic treatment, regardless of the original state of a patient's oral health (Kolip et al., 2016). Daily oral hygiene in orthodontic patients can be more effective if antibacterial mouth rinses are regularly used in addition to brushing and flossing as it has been demonstrated by several clinical trials (Tufekci et al., 2008).

Several modalities of chemical plaque control have been used as adjunctive therapies for treating gingivitis, focusing on proper oral hygiene measures in combination with various dentifrices, gels, and mouthwashes. The most widely used antibacterial mouthwash which is currently considered as the gold standard is chlorhexidine (CHX), an antimicrobial agent that has been proven to reduce levels of microorganisms in the oral cavity (Martin et al., 2016). Although, it is a very compelling product, it presents several side effects associated with its long‐term use. Local side effects such as impaired sense of taste, tooth staining, increased formation of supra‐gingival calculus, occasional irritation and desquamation of mucous membranes have been previously reported (Yeturu et al., 2016).

To overcome these adverse effects, the therapeutic benefits of other natural products, herbs and plant extracts have been investigated in soft tissues. There is a developing body of evidence to suggest that other antioxidants are equally useful in the treatment of gingivitis (Hadj‐Hamou et al., 2020; Scannapieco & Gershovich, 2020). Clinical trials evaluating the use of these antioxidants have shown decreased severity of gingivitis, decreased bleeding on probing, and modest reduction in pocket depths. There are also in vitro and in vivo studies concluding that essential‐oil mouth rinses are capable of eliminating a broad spectrum of microorganisms (Alves et al., 2010; Tufekci et al., 2008). It has been reported that irrigation of gingival pockets with 10% propolis solution decreased gingivitis by 95% suggesting that subgingival irrigation in gingivitis patients can be more effective than scaling, based on clinical and microbiological parameters (Andrade et al., 2017; Coutinho, 2012; Gebaraa et al., 2003).

However, evidence for the use of natural unprocessed products in orthodontic patients is limited and the comparison with a gold standard is missing. The aim of this study is to systematically assess the available data regarding the effects of non‐pharmacological formulations in the treatment of gingivitis during treatment with fixed orthodontic appliances.

## 2 MATERIALS AND METHODS

### 2.1 Protocol and registration

Not available in a public accessible database.

### 2.2 Reporting format

The Preferred Reporting Items for Systematic Reviews and Meta‐Analyses (PRISMA) were adopted throughout the process of the present systematic review (Moher et al., 2009; Moher et al., 2015).

### 2.3 Population (P), intervention (I), comparison (C), outcomes (O), and study design (PICOS)

Participants (Population): Orthodontic patients of any age and sex.

Intervention: Any type of natural and organic products used to control gingivitis. Probiotics or other processed natural products were excluded.

Comparisons: Any control group was accepted.

Outcomes: Quantitative and qualitative analysis of gingival scores or other relevant parameters. Follow‐up: All observation periods were accepted.

Study design: Any study design was considered eligible for inclusion in this review, including randomized controlled trials (RCTs), nonrandomized or quasi‐randomized controlled trials, prospective and retrospective studies.

Exclusion criteria: Animal and in vitro studies. Case reports or studies reporting less than five patients. Studies including patients with systemic disorders affecting periodontal and orthodontic therapy. Preclinical studies/Abstracts/Letters to editors/ Narrative reviews. Insufficient/unclear information not allowing data extraction. No author response to inquiry email for data clarification.

### 2.4 Search strategy

Detailed search strategies were developed and appropriately revised for each database, considering the differences in controlled vocabulary and syntax rules by the last author.

#### 2.4.1 Electronic search

On October 1, 2020 we updated and searched the following electronic databases to find reports of relevant published studies:

- The Cochrane Central Register of Controlled Trials (CENTRAL) (up to October 1, 2020);
- MEDLINE (PubMed) (1946 to September Week 4, 2020);
- Ovid MEDLINE (In‐Process and Other Non‐Indexed Citations, October 1, 2020);
- Ovid EMBASE (1974 to October 1, 2020)
- LILACS (1982 to October 1, 2020)

The search strategy for Medline/PubMed is shown in Table S1.

#### 2.4.2 Unpublished literature search

In order to further identify potential articles for inclusion, gray literature was searched in the register of clinical studies hosted by the U.S. National Institutes of Health (www.clinicaltrials.gov), the multidisciplinary European database (www.opengrey.eu), the National Research Register, and Pro‐Quest Dissertation Abstracts and Thesis databases (https://about.proquest.com).

#### 2.4.3 Manual search

Experts in the field were contacted in order to find additional literature that might be relevant. The reference lists of all identified eligible studies and other published systematic reviews were handsearched in order to identify further eligible studies. No language or publication time restrictions were applied.

### 2.5 Study selection

Study selection was performed independently and in duplicate by the first two authors of the review, who were not blinded to the identity of the authors of the studies, their institutions, or the results of their research. Study selection procedure comprised of title‐reading, abstract‐reading and full‐text‐reading stages. After exclusion of noneligible studies, the full report of publications, considered by either author as eligible for inclusion, was obtained and assessed independently. Disagreements were resolved by discussion and consultation with the third author of the review. A record of all decisions on study identification was kept.

### 2.6 Data collection

The first two authors performed data extraction independently and in duplicate. Disagreements were resolved by discussion with the last author. Specifically designed excel collection forms were used to record the desired information. If stated, the sources of funding, trial registration, and publishing of the trial's protocol was recorded. This information was used to aid assessment of heterogeneity and the external validity of the included studies. In case of missing data, it was attempted to contact the corresponding author.

### 2.7 Quality assessment

The methodological quality of all included studies was assessed by the first two review authors, independently and in duplicate. For interventional, randomized controlled trials (RCTs) the Risk of Bias 2.0. tool was used (Sterne et al., 2019).

### 2.8 Data analysis

Meta‐analyses would have been conducted and pooled estimates would have been calculated if studies with similar comparisons reported the same outcomes, with similar setup and follow‐up.

### 2.9 Heterogeneity

Clinical and methodological heterogeneity were assessed by examining the characteristics of the studies, the similarity between the types of participants, the interventions, and the outcomes as specified in the inclusion criteria for considering studies for this review. Statistical heterogeneity would have been assessed using a Chi^2^ test and the *I* ^2^ statistic.

### 2.10 Assessment of reporting bias

Reporting biases arise when the reporting of research findings is affected by the nature or direction of the findings themselves. Potential reporting biases including publication bias, multiple (duplicate reports) publication bias and language bias in this review, were reduced by conducting an accurate and at the same time a sensitive search of multiple sources with no restriction on language. A search for ongoing trials was conducted too. In the presence of more than 10 studies in a meta‐analysis, the possible presence of publication bias would have been investigated for the primary outcome.

### 2.11 Subgroup analyses

If there was sufficient data, subgroup analyses would have been conducted to explore the influence of study characteristics such as gender and/or jaw.

### 2.12 Sensitivity analysis

We intended to explore whether or not the analysis of studies stratified by design or by risk of bias (i.e., overall low risk versus high risk) yielded similar or different results.

### 2.13 Unit of analysis issues

We anticipated that some of the included studies presented data from repeated observations on participants, which could lead to unit‐of‐analysis errors. In such cases, we followed the advice provided in section 9.3.4 of the Cochrane Handbook for Systematic Reviews of Interventions (Higgins & Green, 2011).

## 3 RESULTS

### 3.1 Description of the included studies

**FIGURE 1:**
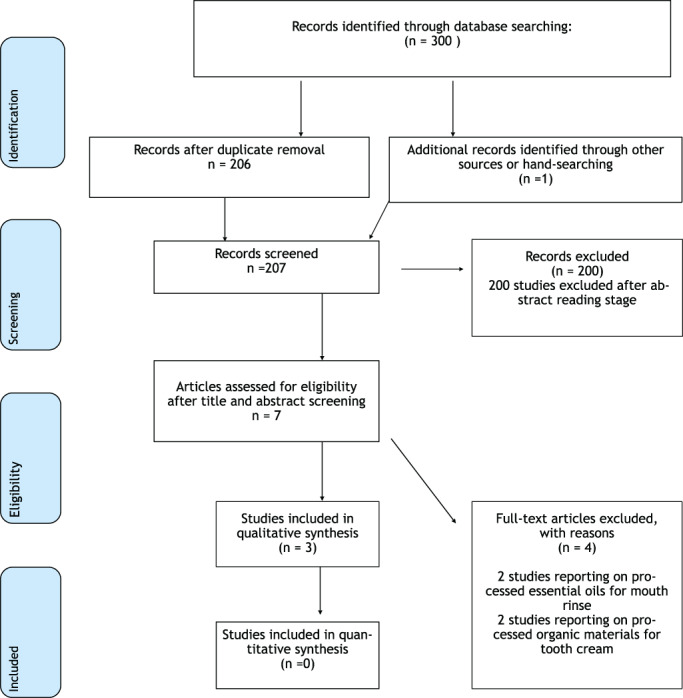
Flow diagram of studies' inclusion

The flow diagram of study selection is shown in Figure 1. A total of 300 studies were initially identified in the electronic search. After exclusion of duplicates and title and abstract screening, seven studies were retrieved to be examined in more detail. Four studies were subsequently excluded after full text assessment, leaving three studies eligible for inclusion (Albuquerque et al., 2010; Andrade et al., 2017; Santos et al., 2003).

All included studies were RCTs. An overview of main characteristics of the included studies is presented in Table 1. A total 135 patients were examined with the sample size varying from 20 to 85 participants with an age range between 12 and 40 years. Treatment duration and therefore follow‐up period varied from 30 min to 15 days. Control groups used either a placebo mouthwash (Andrade et al., 2017) or a choice from a variety of mouthwashes, such as CHX (Albuquerque et al., 2010; Andrade et al., 2017), sucrose or sorbitol solutions (Santos et al., 2003), and chlorine dioxide mouth rinse (Albuquerque et al., 2010). The effect of the organic agents used in treating gingivitis during treatment with fixed orthodontic appliances was assessed by means of plaque indices (PI), gingival indices (GI), PH of plaque collection, and bacterial counts.

**TABLE 1:** Characteristics of included studies

Study	Study design	Aim	Sample	Age	Treatment	Control	Treatment duration	Follow‐up	Method of outcome assessment
Goes et al. (2016)	RCT	To evaluate the effects of a mouthwash containing Matricaria Chamomile extract for orthodontic patients with gingivitis.	30 (4 M, 26 F)	Mean age, 28.8 ± 3.28 years	15 ml of 1% Matricaria Chamomile L. Mouthwash twice daily	Group A: 15 ml of 0.12% CHX twice daily Group B: 15 ml of placebo twice daily	15 days	Day 1Day 15	‐ Visible plaque Index (VPI) ‐ Gingival Bleeding Index (GBI)
Yeturu et al. (2016)	RTC	To evaluate the effect of *Aloe vera*, chlorine dioxide, and chlorhexidine mouthrinses on plaque and gingivitis during orthodontic treatment	85 (40 M,45F)	Mean age = 21.53 ± 3.41 years	Aloe vera mouth rinse (10 ml for 1 min twice daily)	Group A: CHX mouth rinse (10 ml for 1 mm twice daily) Group B: Chlorine dioxide mouth rinse (10 ml for 1 min twice daily)	15 days	15 days	‐ Modified Silness and Loe Plaque Index ‐ Gingival Index
Atwa et al. (2014)	RCT	(a) To determine the effect of chewing honey on plaque pH and bacterial counts present in dental plaques (b) To determine the in vitro effects of honey on the growth of plaquebacteria.	20 F	Age range: 12–18 years	Chew and ingest 10 gr of pure undiluted honey in 2 min	15 ml of 10% sucrose solution (positive control) or 10% sorbitol solution (negative control) for 1 min	30 min	2, 5, 10, 20, 30 min	‐ PH of plaque collection ‐ bacterial counts

Abbreviations: F, females; M, males; RCT, randomized clinical trial; min, minute.

### 3.2 Quality assessment

An overview of the risk of bias assessment is given in Table 2. All included studies were rated at high risk of bias (Albuquerque et al., 2010; Andrade et al., 2017; Santos et al., 2003). Major concern across studies was the lack of exact information about randomization process and the absence of assessor blinding.

**TABLE 2:** Quality assessment of included randomized clinical trials (RCTs)

Study	Bias arising from the randomization process	Bias due to deviations from the intended interventions	Bias die to missing outcome data	Bias in measurement of the outcome	Bias in selection of the reported result	Overall bias
Goes et al. (2016)	Authors' judgment: Some concerns Support for judgment: Insufficient information about the sequence generation but allocation concealment properly performed	Authors' judgment: Low Risk Support for judgment: Blinding of participants and personnel achieved	Authors' judgment: Low Risk Support for judgment: All outcome data available	Authors' judgment: High Risk Support for judgment: Outcome assessors not blinded	Authors' judgment: Low Risk Support for judgment: Reported outcome data unlikely to have been selected.	Authors' judgment: High risk
Yeturu et al. (2016)	Authors' judgment: Low Risk Support for judgment: Sufficient information about the sequence generation and allocation concealment	Authors' judgment: High Risk Support for judgment: Insufficient information about the blinding of participants and personnel	Authors' judgment: Low Risk Support for judgment: All outcome data available	Authors' judgment: High Risk Support for judgment: Outcome assessors not blinded	Authors' judgment: Low Risk Support for judgment: Reported outcome data unlikely to have been selected.	Authors' judgment: High risk
Atwa et al. (2014)	Authors' judgment: Some concerns Support for judgment: Method of randomization and allocation concealment not clearly reported	Authors' judgment: High Risk Support for judgment: Insufficient information about the blinding of participants and personnel	Authors' judgment: Low Risk Support for judgment: All outcome data available	Authors' judgment: High Risk Support for judgment: Outcome assessors not blinded	Authors' judgment: Low Risk Support for judgment: Reported outcome data unlikely to have been selected.	Authors' judgment: High risk

### 3.3 Qualitative synthesis of results

The results of the included studies are presented in Table 3. The diversity between the composition of the products, the treatment duration, the frequency of use, the follow‐up of the study, the controls and the outcomes did not lead to studies with comparable outcome measures. Therefore, methodological and clinical heterogeneity precluded a quantitative synthesis of the results.

**TABLE 3:** Results of included studies

Study	Method of outcome assessment	Results	Conclusions
Goes et al. (2016)	Visible plaque Index (VPI) Gingival Bleeding Index (GBI)	Placebo Group: increase in VPI and GBI (10.2% and 23.1, respectively) from day 1 to day 15 MTC Group: decrease in VPI and GBI (−25.6% and − 29.9% respectively) from day 1 to day 15 CHX Group: decrease in VPI and GBI (−39.9% and − 32.0% respectively) from 1 day to day 15	MTC reduced biofilm accumulation and gingival bleeding in patients with gingivitis and did not cause side effects associated with CHX
Yeturu et al. (2016)	Modified Silness and Loe Plaque Index Gingival Index	Mean percentage reduction of PI: (a) aloe vera = 20.38 ± 16.74 (b) CHX = 31.59 ± 16.58 (c) chlorine dioxide = 30.29 ± 18.30 mean percentage reduction of GI: (a) aloe vera = 9.88 ± 8.77 (b) CHX = 16.30 ± 9.98 (c) chlorine dioxide = 12.22 ± 9.30	Chlorine dioxide can be a suitable and economical alternative for chlorhexidine. Aloe vera was not equally effective.
Atwa et al. (2014)	PH of plaque collection Bacterial counts	(a) The pH observed for the sorbitol group did not change over time (b) Bacterial counts were significantly reduced in the honey group compared to the other treatment groups (c) honey significantly inhibited the growth of all studied strains compared to inhibition observed with antibiotics	Topical application of honey can modify the pH, reduce bacterial counts and inhibit bacterial growth

### 3.4 Plaque index and gingival index

Two studies evaluated the effects of non‐pharmacological formulations in plaque index (PI) and gingival index (GI, Albuquerque et al., 2010; Andrade et al., 2017). Both indices decreased throughout various follow‐up times (Table 3).

In the *Aloe Vera* group a reduction of 20.38% and 9.88% in plaque and gingival index scores was recorded, respectively (Albuquerque et al., 2010).

### 3.5 PH and bacterial counts

One study evaluated the effects of non‐pharmacological formulations in the PH of the oral cavity and demonstrated that honey can modify PH, decrease bacterial counts and prevent bacterial growth (Table 3). It was found that chewing honey decreased PH as early as 5 min after initiation and recovered it after 20 min. It should be reported though that throughout the study duration (30 min) it never dropped below the critical value of decalcification (PH = 5,5) (Santos et al., 2003).

## 4 DISCUSSION

Gingivitis and its treatment in orthodontic patients have always been an issue and a challenge for clinicians. The presence of fixed orthodontic appliances and archwires make mechanical plaque removal more difficult (Yeturu et al., 2016) and daily oral hygiene time‐consuming (Tufekci et al., 2008). Plaque is easily accumulated around brackets, bands, wires, and ligatures causing subsequent gingivitis. Clinical trials have shown that oral health status is significantly improved when antibacterial mouth rinses are added to the daily oral hygiene regimen with tooth‐brushing and flossing (Santos et al., 2003).

The most frequently used and well‐known antiseptic mouthwash is chlorhexidine, with a broad bactericidal and bacteriostatic spectrum due to its binding properties accompanied by a high substantivity of up to 12 h within the oral cavity (Goes et al., 2016). Although, its systemic toxicity is small due it is poor absorption in the gastrointestinal tract, several side effects have been reported and therefore increased consideration has been given to other antimicrobial products.

Non‐pharmacological formulations manage to control gingivitis owing to their various components and their mechanisms of action. Particularly, antioxidant essential oil gel has been effective in reducing plaque and gingival inflammation levels because of the antioxidant component of the gel which produces anti‐inflammatory interleukins and reduces inflammatory mediators. There are in vivo studies that have recorded improvement in BOP and GI that has been attributed to the essential oil component (Gunsolley, 2010; Tufekci et al., 2008; Van Leeuwen et al., 2011).

Moreover, as previous and present studies have shown the effect of 1% MTC mouthwash can be attributed to its immune‐modulatory activity. MTC extracts (flavonoid apigenin) and its terpenic derivatives (chamazulene, β‐bisabolol, and A and 2B bisabolol‐oxides) have significant anti‐inflammatory and antioxidant activities and sufficiently contribute to gingival inflammation reduction and control of peri‐implantitis. A study on oral biofilm reported that an MTC extract was effective against *Staphylococcus aureus* and Candida (Nogueira et al., 2008) and inhibited growth of Streptococcus Mutans and Streptococcus Sanguinis, important initial colonizers (Albuquerque et al., 2010). In the study by Goes et al. although VPI and GBI were significantly decreased in participants receiving a 1% MTC mouthwash, they did not differ when compared to those receiving a CHX mouthwash (Goes et al., 2016).

The reduction that *Aloe Vera* stimulates in plaque and gingival indices in orthodontic patients is likely on the grounds of its active compounds like aloesin, aloin, aloeride, flavonoids, saponin and sterols, which have antibacterial, anti‐inflammatory and antioxidant properties. Likewise, chlorine dioxide has the same properties by inactivating enzymes, misbalancing electrolytes within cell membranes and disrupting protein synthesis. It has also been found to oxidize VSCs, components responsible for inflammation and disease progression (Yeturu et al., 2016). The mechanism associated with antibacterial effects of honey continues to be unknown, though the presence of hydrogen peroxide, flavonoids and hypertonic sugar concentration tend to be the most likely factors.

A major limitation of all included studies, and consequently of the current review, is that recruited patients did not follow the same dental hygiene protocol and, furthermore, the lack of information on participants' compliance with home‐care oral hygiene regimens that may have altered the overall effect of the antimicrobial used. In previous studies compliance has been found to range between 68% and 82%, with self‐reporting compliance being overestimated. Studies on predicting factors affecting patient's compliance reported that cooperation varied depending on the patient's age and sex, perception of malocclusion, and socioeconomic factors (Tufekci et al., 2008). The use of a written reporting system with periodic reminders to the participants might be useful and actually increase actual compliance, providing a better estimate of the true effect size.

## 5 CONCLUSIONS

- Non‐pharmacological formulations reduced biofilm accumulation and gingival indices in orthodontic patients with gingivitis.
- Their effect is attributed to their antimicrobial and anti‐inflammatory activities.
- No reports on any side effects similar to those associated with CHX.

## CONFLICT OF INTEREST

The authors declare to have no commercial relationship or conflict of interest with any of the products used in this investigation and designed the study at their own initiative. This study was self‐funded by the authors.

## AUTHORS' CONTRIBUTIONS

All authors have made substantial contributions to conception and design of the study. Chrysanthi Papadopoulou, Ioanna Karamani and Sofia Gkourtsogianni have been involved in data collection, quality assessment and data analysis. Dimitrios Kloukos and Kiriaki Seremidi have been involved in data interpretation, drafting the manuscript and revising it critically and have given final approval of the version to be published.

## Supporting information


**Table S1:** PubMed Search Strategy

## Data Availability

Data available on request from the authors.
