# Supplementary material for: A systematic review on the effectiveness of organic unprocessed products in controlling gingivitis in patients undergoing orthodontic treatment with fixed appliances
Source: Clin Exp Dent Res. 2021 May 4;7(5):664–71. doi: 10.1002/cre2.417 (PMC8543457; doi:10.1002/cre2.417)
Supplement: Supplementary file 1 — Table S1: PubMed Search Strategy [file CRE2-7-664-s001.pdf]

PubMed Search Strategy

Search number	Query	Results
1	(gingivitis) AND (orthodontics)	3,936
2	(gingivitis[Title/Abstract]) AND (orthodont*[Title/Abstract])	246
3	(gingivitis[MeSH Terms]) AND (orthodont*[Title/Abstract])	320
4	((gingivitis[Title/Abstract]) AND (orthodont*[Title/Abstract])) AND (non-pharmac*)	0
5	((gingivitis[MeSH Terms]) AND (orthodont*[Title/Abstract])) AND (pure[Title/Abstract] OR natural[Title/Abstract] OR organic OR[Title/Abstract])	6
6	((gingivitis[MeSH Terms]) AND (orthodont*[Title/Abstract])) AND (unprocessed)	0
7	(periodontal health[Title/Abstract]) AND (orthodont*[Title/Abstract])	258
8	((periodontal health[Title/Abstract]) AND (orthodont*[Title/Abstract])) AND (pure[Title/Abstract] OR natural[Title/Abstract] OR organic[Title/Abstract])	5
